# Ciclosporin-Induced Generalised Hypertrichosis in a Transgender Woman Receiving Testosterone Blockers

**DOI:** 10.7759/cureus.103296

**Published:** 2026-02-09

**Authors:** Zoe I Hemsley, Andre Khoo

**Affiliations:** 1 Dermatology, University of Cambridge, Cambridge, GBR; 2 Dermatology, Addenbrooke's Hospital, Cambridge University Hospitals NHS Foundation Trust, Cambridge, GBR

**Keywords:** ciclosporin, cyclosporine, dermatology, drug side-effect, hypertrichosis, plaque psoriasis, testosterone

## Abstract

Hypertrichosis is a well-documented side effect of ciclosporin, yet it is rarely encountered in dermatological practice. We present a case of hypertrichosis in a transgender woman on testosterone-blocking therapy, illustrating that ciclosporin can induce hypertrichosis even in the context of androgen suppression.

A 25-year-old transgender woman with severe plaque psoriasis developed generalised hypertrichosis after commencing ciclosporin. She was concurrently taking estradiol and triptorelin as part of gender-affirming therapy. Hypertrichosis involved the arms, fingers, and face, leading to distress and subsequent discontinuation of ciclosporin. The patient was transitioned to adalimumab, which achieved good disease control without recurrence of hypertrichosis.

This appears to be the first documented case of ciclosporin-induced hypertrichosis in a transgender woman receiving testosterone blockers. Clinicians should remain mindful of this potential adverse effect and its psychological impact when prescribing ciclosporin to transgender individuals. Further research is warranted to clarify the role of sex hormones in susceptibility to this side effect.

## Introduction

Hypertrichosis is a well-documented but uncommon side effect of ciclosporin, most often reported in patients receiving long-term immunosuppressive therapy. Ciclosporin is an immunomodulatory agent used in dermatology for short-term management of severe inflammatory dermatoses, including plaque psoriasis, due to its rapid immunosuppressive effects [1]. Here, we present a case of hypertrichosis in a transgender woman taking testosterone blockers. Whether hormonal therapies potentiate the risk of this side effect is currently unknown, and clinicians should remain mindful of the potential for hypertrichosis in transgender patients and the associated psychological distress. Further research is warranted to clarify whether sex-hormone milieu modulates susceptibility.

Hypertrichosis is a disorder of excessive hair growth in a normal distribution, in areas not usually associated with androgen activity, as opposed to hirsutism, which describes hair growth in a stereotypically male pattern [2]. The causes of hypertrichosis are broad, and include associations with conditions such as underlying malignancy, malnutrition, and porphyria cutanea tarda, as well as side effects of drugs, for instance androgenic steroids, phenytoin, and ciclosporin. Infrequently, the phenomenon can be congenital, as a result of a genetic mutation [3]. In contrast, transgender women taking androgen-blocking therapy and oestrogen experience a significant reduction in terminal hair growth, six to 12 months after starting treatment [4].

Whilst hypertrichosis is a known side effect of ciclosporin, it is infrequently seen in dermatological practice, affecting only 6% of patients [5]. When it is seen, it is believed to be in a dose-dependent manner, with hair first appearing on average four to eight weeks after initiation [6].

We believe this is the first documented case of hypertrichosis in a transgender woman. A targeted literature search was conducted in PubMed and Google Scholar (last searched October 2025). No prior reports of ciclosporin-induced hypertrichosis in transgender patients were identified.

## Case presentation

The patient initially started taking gender-affirming medications in December 2022, with a 100mg Estraderm patch, containing 3.0mg estradiol, daily. In May 2023 she started taking triptorelin, a gonadotropin-releasing hormone (GnRH) agonist, 3.0mg intramuscularly, every four weeks. GnRH agonists work by eliciting an initial surge of gonadotropin release, which subsequently results in down-regulation of gonadotropin synthesis, and therefore reduced production of testosterone [7]. Prior to the episode of ciclosporin-induced hypertrichosis, she used hair removal methods such as plucking and home intense pulsed light (IPL) therapy. Hair is an important part of personal and gender identity, and hair removal can be an important part of transitioning for female transgender patients [8].

The patient had a diagnosis of severe chronic plaque psoriasis with a baseline Psoriasis Area and Severity Index (PASI) of 27.6 and a Dermatology Life Quality Index (DLQI) of 17/30. She had responded well to treatment with methotrexate, but this was discontinued following persistently mild transaminitis (ALT). She had no other comorbidities, and was not taking any other regular medication at the time of presentation. Following this, she was switched to ciclosporin, 2.5mg/kg o.d. on 3rd January 2024. Serum ciclosporin levels were not routinely measured, as this was not indicated given the stable treatment course; renal function and blood pressure monitoring remained normal throughout. On follow-up three months after initiation, her skin was almost clear with minimal residual disease. However, she reported a significant degree of hypertrichosis. She described generalised hair growth including areas that were not previously a problem, such as the arms, fingers, and face (non-androgenic sites). She first started noticing the gradual hair growth approximately three to four weeks into treatment, which increased throughout February and March, consistent with other reports of ciclosporin-induced hypertrichosis in the literature. The excess hair was darkly pigmented, with increased thickness at the base and a finer distal taper, suggestive of vellus-to-terminal transformation. The density was evenly distributed, involving more than half of the existing hair follicles. There were no clinical features or laboratory findings suggestive of alternative causes of hypertrichosis, and no history of malignancy, endocrine disease, malnutrition, or porphyria cutanea tarda. She described the experience as very stressful, and experienced grief when seeing herself in the mirror. She sought electrolysis privately as treatment for this, and her hair returned to normal in late May 2024.

As a result, ciclosporin was discontinued and she switched to adalimumab, which proved to be a successful treatment. The treatment timeline is shown in Table 1.

**Table 1:** Timeline of Treatment and Key Clinical Events GnRH: gonadotropin-releasing hormone, ALT: alanine aminotransferase

Date	Event	Notes
14 December 2022	Initiated estradiol patch	Estraderm 100 mg patch (3.0 mg estradiol) applied daily as part of gender-affirming hormone therapy. Ongoing at time of report.
26 May 2023	Initiated GnRH agonist	3.0 mg triptorelin intramuscular injection every four weeks for testosterone suppression. Ongoing at time of report.
03 November 2023	Initiated methotrexate	2.5mg every week. Used for severe chronic plaque psoriasis.
03 January 2024	Discontinued methotrexate	Discontinued following persistent mild transaminitis (ALT).
03 January 2024	Initiated ciclosporin	2.5 mg/kg daily for psoriasis. Renal function and blood pressure remained normal.
Late January 2024 (~3-4 weeks after cyclosporine initiation)	Onset of hypertrichosis	Gradual hair growth noted on face, arms, and fingers (non-androgenic sites), progressively increasing over the following weeks and months.
30 January - 24 April 2024	Series of electrolysis sessions	Undertaken privately to address hypertrichosis. Sessions on Jan 30, Feb 14, Feb 27, Mar 12, and Apr 24.
09 April 2024	Discontinued ciclosporin	Stopped due to distress from hypertrichosis.
Late May 2024	Resolution of hypertrichosis	Hair growth resolved following discontinuation of ciclosporin.
03 September 2024	Initiated adalimumab	40mg subcutaneous injection every 2 weeks. Switched for psoriasis management. Achieved good disease control without recurrence of hypertrichosis. Ongoing at time of report.

## Discussion

Ciclosporin is an immunomodulatory agent licensed to treat severe psoriasis where conventional therapies, such as corticosteroids and vitamin D analogues, have been ineffective [1]. Ciclosporin predominantly exerts its immune-modulating effects by inhibiting the production of cytokines involved in T-cell activation regulation, particularly interleukin-2 [9]. At the time of writing, there is currently no definitive answer on the mechanism of ciclosporin-induced hypertrichosis. Indeed, it is not entirely understood how the process of hair cycling occurs [10].

Several theories have been proposed to explain the relationship between ciclosporin and hypertrichosis [11]. According to Xu et al., 2012 [12], the effect is testosterone-independent, mediated by ciclosporin increasing growth factors such as vascular endothelial growth factor (VEGF), and promoting the proliferation of matrix cells. Whereas, according to Ponticelli et al. (2011), the proposed mechanism of action for ciclosporin-induced hypertrichosis is that ciclosporin induces alpha-reductase activity, an enzyme responsible for converting androgens to dihydrotestosterone (DHT) [13]. Indeed, DHT is implicated in both hirsutism and androgenetic alopecia [14]. It is possible that ciclosporin influences multiple pathways involved in hair follicle cycling.

Further study is warranted to determine whether variations in sex-hormone milieu have a modifying role in ciclosporin-induced hypertrichosis. However, current evidence suggests that this adverse effect is not influenced by systemic androgen levels. While ciclosporin-induced hair growth may appear more pronounced in women with darker hair due to greater contrast, its prevalence does not differ significantly between sexes [5]. It is, however, more frequently reported in children [15]. Transgender women receiving estradiol and GnRH agonists typically achieve serum androgen levels comparable to cisgender women [4], making androgen suppression alone an unknown explanatory factor in this case.

The occurrence of hypertrichosis in a transgender woman under androgen suppression therapy is clinically noteworthy because it demonstrates a non-androgenic pattern of hair growth, contrasting with the expected reduction in hair from hormone therapy. Transgender women taking oestrogen and testosterone blocking therapy can reliably expect a reduction in hair growth six to 12 months after medication initiation [4]. Importantly, ciclosporin does not raise systemic androgen levels; in fact, animal models have demonstrated decreased serum testosterone during treatment [16]. This further supports the likelihood of non-androgenic mechanisms contributing to the observed phenomenon.

## Conclusions

This case represents, to our knowledge, the first documented instance of ciclosporin-induced generalised hypertrichosis in a transgender woman receiving testosterone-blocking therapy. Clinicians prescribing ciclosporin to transgender patients receiving hormonal therapy should remain mindful of this potential adverse effect and its associated psychological impact. Further studies are warranted to better understand how variations in the hormonal milieu may influence susceptibility to this side effect.

Written informed consent was obtained from the patient for publication of this case report and accompanying details. The patient declined to provide clinical photographs due to the distressing nature of the case, and this decision was fully respected.
